# *Actinidia chinensis* Planch.: A Review of Chemistry and Pharmacology

**DOI:** 10.3389/fphar.2019.01236

**Published:** 2019-10-30

**Authors:** Xirui He, Jiacheng Fang, Xufei Chen, Zefeng Zhao, Yongsheng Li, Yibing Meng, Linhong Huang

**Affiliations:** ^1^Department of Bioengineering, Zhuhai Campus Zunyi Medical University, Zhuhai, China; ^2^The College of Life Sciences, Northwest University, Xi’an, China; ^3^Honghui Hospital, Xi’an Jiaotong University, Xi’an, China

**Keywords:** *Actinidia chinensis*, nutritional composition, chemistry, pharmacological properties, antitumor, antioxidant

## Abstract

*Actinidia chinensis* Planch. (*A. chinensis*), commonly known as Chinese kiwifruit, is a China native fruit, which becomes increasingly popular due to attractive economic, nutritional, and health benefits properties. The whole plant including fruits, leaves, vines, and roots of *A. chinensis* are used mainly as food or additive in food products and as folk medicine in China. It is a good source of triterpenoids, polyphenols, vitamin C, carbohydrate, amino acid, and minerals. These constituents render the *A. chinensis* with a wide range of pharmacological properties including antitumor, antioxidant, anti-inflammatory, immunoregulatory, hypolipemic, antidiabetic, and cardiovascular protective activities, suggesting that it may possibly be value in the prevention and treatment of pathologies associated to cancer, oxidative stress, and aging. This minireview provides a brief knowledge about the recent advances in chemistry, biological activities, utilization, and storage of Chinese kiwifruit. Future research directions on how to better use of this crop are suggested.

## Introduction

**Figure 1:**
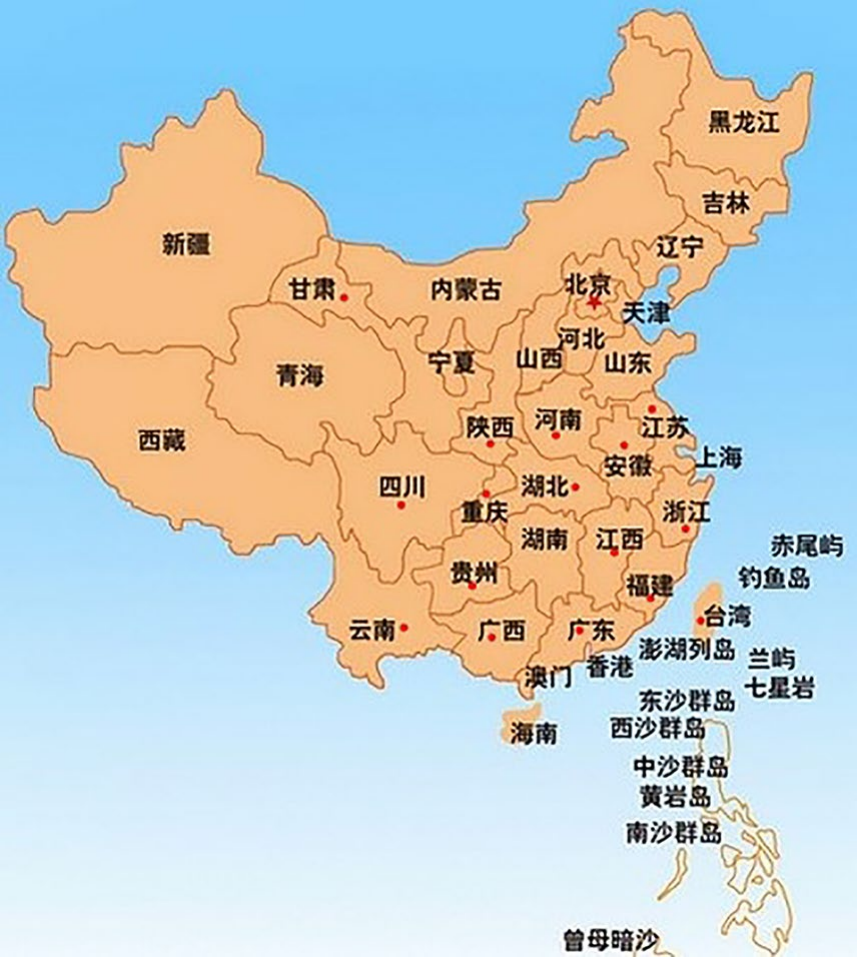
The red spots in the map depicted the main region of *A. chinensis* distribution in China (Flora of China, 2007; https://www.newasp.net/soft/105257.html).

*Actinidia chinensis* Planch. (*A. chinensis*), commonly known as “Chinese kiwifruit” (English), “中华猕猴桃” (Chinese), and characterized by excessive vegetative vigor, is a woody perennial, deciduous, and functionally dioecious medicinal plant in the family Actinidiaceae (Flora of China, 2007; The Plant List, 2013). It is native to China and has been cultivated in New Zealand, United States, Greece, Italy, Chile, France, Japan, and Korea (Li and Zhu, 2017; Ma et al., 2017). In China, they are mainly distributed in temperate to warm-temperate zones such as Shaanxi, Gansu, Henan, Guangdong, Guangxi, Fujian, Guizhou, Yunnan, Sichuan, as well as the middle and lower reaches of the Yangtze River basin, especially in Yiling district in Yichang city, Hubei province (**Figure 1**) (Flora of China, 2007). There are 13 *A. chinensis* cultivars, especially “Hongyang,” “Jintao,” and “Huayou,” are developed for commercial production in China (Sharon, 2016), and more than three ones such as “Sungold,” “Charm,” and “Hort16A” developed in New Zealand (Henare, 2016) (**Table 1**).

**Table 1:** *A. chinensis* cultivars developed for commercial production (Henare, 2016).

Origin country	Cultivar	Fruit shape	Avg. weight	Fruit skin	Fruit ﬂesh
China	Cuiyu (Liangmei No. 1)	Ovoid	90 g	Greenish brown with short hairs	Green
	Wuzhi No. 3 (Wuzhi 81-36)	Ellipsoid	85 g	Dark green with soft hairs	Bright green
	Chuhong (Panda™ Forest Red Kiwi)	Long ellipsoid	80 g	Dark green and hairless	Green with red flesh around white core
	Qihong	Cylindric	100 g	Green with sparse or absent hairs	Light green to yellow
	Hongyang (Red Sun, RS1)	Obovoid	60-70 g	Dark green or greenish brown with fine hairs	Green-yellow to yellow, circle of red around white core
	Jintao (C6, WIB-C6, Jingold™)	Long cylindric	90 g	Yellow with brown hairs	Green-yellow to orange-yellow
	Huayou (Panda™ Golden Kiwi)	Ellipsoid	90 g	Ellipsoid	Light green to yellow
	Ganmi No. 1 (Zaoxian No. 1, FT-79-5)	Cylindric	85 g	Green-brown to pale brown with soft hairs	Greenish-yellow to yellow
	Ganmi No. 3 (Jinfeng, FT 79-3)	Ellipsoid	80-90 g	Yellow-brown or dark brown with short, fine hairs	Yellow
	Jinyan	Cylindrical	100-110 g	Yellow brown with short, fine hairs	Yellow
	Ganmi No. 2 (Kuimi, FY 79-1)	Apple shaped	100 g	Green-brown to dark brown with fine hairs	Yellow-green to yellow
	Hort16A	Ovoid	95-100g	Green-brown to brown with soft hairs	Yellow-green to bright yellow
	Wanhong	Cylindrical	110-140g	Green-brown with rare hairs	Yellow-green to bright yellow
New Zealand	Charm (Zespri^®^ Charm)	Ovoid		Brown with soft hairs	Yellow
	Sungold (Zespri^®^ Sungold)			Brown with smooth skin	Yellow
	Hort16A (Zespri^®^ Gold, Earligold)	Ovoid	95-100g	Green-brown to brown with soft hairs	Yellow-green to bright yellow
Italy	Soreli (Ac 171.76)	Oblong	> 100g	Brown with sparse hairs	Yellow
Japan	Sanuki Gold	Squat	160-180g	Brown with soft hairs	Bright yellow

**Figure 2:**
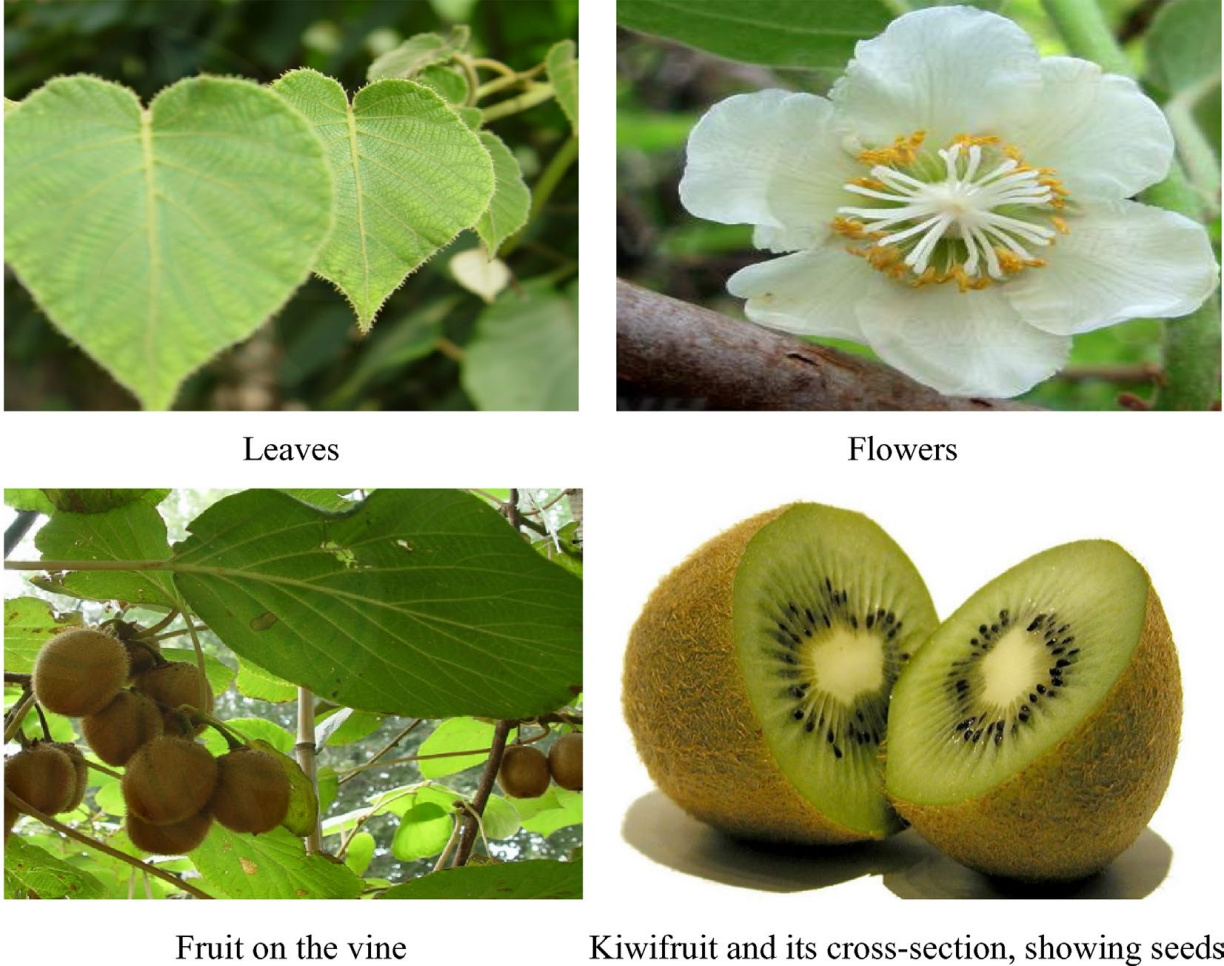
The leaves, flowers, vines, and fruits of *A. chinensis*.

There are two varieties accepted by *The Plant List* that include *A. chinensis* and *A. chinensis* var. *setosa* H.L.Li (The Plant List, 2013). The fruit of *A. chinensis* is the largest one in *Actinidia* genus, and it has the greatest economic, medicinal, and edible significance in terms of production and utilization. Its relevant pictures are showed in **Figure 2**. Generally, Chinese kiwifruit with a cross-sectional radius of about 3 cm is oval-shaped densely covered with yellowish-brown hairs. The flesh color of fruit skin is green to yellow, and the average fruit weight is 20–120 g. The fruit is a tasty, nutritious food that can be eaten fresh directly. Today, a range of kiwifruit processed products with the attractive eating quality and nutritional benefits has been developed including juice, preserved fruit, yogurt, wine, canned fruit, dried kiwi slices, fruit vegetable juice drinks, milk beverage, and vinegar. Apart from being a food and natural health product, the whole plant (fruits, branches and leaves, vines and roots) of *A. chinensis* has been used as traditional folk medicine in China (He et al., 2017; Wei et al., 2018; Fang et al., 2019). The ripe kiwifruit, tastes sweet and sour, acts on the spleen, stomach, and kidney meridians, has improving properties on dyspepsia, loss of appetite, and vomiting. The branches and leaves have been used to treat arthronalgia, bleeding, empyrosis, and ulcer. The vine has appetizing, heat clearing, and wind-dampness dispelling effects and is used to treat indigestion, aundice, and urolithiasis. The root and bark of *A. chinensis* taste bitter and astringent, and they have various medical effects such as wind and heat dispelling, blood circulation improving, and detumescence properties, and are used for the treatment of rheumatoid arthritis, bruises, furuncle, swelling, filariasis, hepatitis, and dysentery (Xie, 1975). However, people with weak spleen and stomach should be cautious in taking *A. chinensis* (Xie, 1975). To date, only very few modern studies have been done on potential toxic and side effects of *A. chinensis*, which should be highlighted in future research.

The principal chemical composition of the whole plant of *A. chinensis* include polyphenol, triterpenoids and derivatives, carotenoids, polysaccharides, amino acids, vitamins, essential oils, and microelements. (Papunidze et al., 2001; Chang and Case, 2005; Ma et al., 2017; Wang et al., 2017; Twidle et al., 2018). Among these ingredients, the main bioactive constituents are phenolic compounds, triterpenes, and the major nutritional composition are vitamin C, vitamin E, dietary fiber, and microelements, which make up a relatively significant share of the daily value (**Table 2**). Pharmacological results have revealed various promising bioactivities to *A. chinensis* including antitumor, antioxidant, anti-inflammatory, antimicrobial, immunoregulatory, hypolipemic and antidiabetic, cardiovascular protective, hypnotic effects, and ACE inhibitory activities (Deng et al., 2013; Niu et al., 2016; Sun et al., 2017; Xia et al., 2017; Deng et al., 2018; Hou et al., 2018; Fang et al., 2019). Much of these bioactivities of *A. chinensis* are consistent with those observed in traditional folk medicine. More importantly, *A. chinensis* showed significantly antitumor and antioxidant properties, and these effects could be depended on the presence of a range of triterpenoids, polysaccharide, and phenolic compounds (Chang and Case, 2005; Wei et al., 2018; Fang et al., 2019). However, the information on the chemical and biological activities of *A. chinensis* is scattered. In this review, we intend to systematically summarize the recent advances in nutritional composition, chemistry, and biological activities of *A. chinensis* and also provide future research directions for better utilize and develop it as a sustainable crop.

**Table 2:** Nutritional composition of Zespri^®^ sun-gold kiwifruit.

Nutrient	Unit	Kiwifruit 81g	Value per 100 g
Proximates			
Water	g	66.78	82.44
Energy	kcal	51	63
Protein	g	0.83	1.02
Total lipid (fat)	g	0.23	0.28
Carbohydrate, by difference	g	12.79	15.79
Fiber, total dietary	g	1.1	1.4
Sugars, total	g	9.96	12.3
Minerals			
Calcium, Ca	mg	14	17
Iron, Fe	mg	0.17	0.21
Magnesium, Mg	mg	10	12
Phosphorus, P	mg	20	25
Potassium, K	mg	255	315
Sodium, Na	mg	2	3
Zinc, Zn	mg	0.06	0.08
Vitamins			
Vitamin C, total ascorbic acid	mg	130.7	161.3
Thiamin	mg	0.000	0.000
Riboflavin	mg	0.060	0.074
Niacin	mg	0.187	0.231
Vitamin B-6	mg	0.064	0.079
Folate, DFE	µg	25	31
Vitamin B-12	µg	0.06	0.08
Vitamin A, RAE	µg	1	1
Vitamin A, IU	IU	19	23
Vitamin E (alpha-tocopherol)	mg	1.13	1.40
Vitamin D (D2 + D3)	µg	0.0	0.0
Vitamin D	IU	0	0
Vitamin K (phylloquinone)	µg	4.9	6.1
Lipids			
Fatty acids, total saturated	g	0.053	0.065
Fatty acids, total monounsaturated	g	0.019	0.023
Fatty acids, total polyunsaturated	g	0.090	0.111
Fatty acids, total trans	g	0	0
Cholesterol	mg	0	0

Source: USDA Food Composition Databases, https://ndb.nal.usda.gov/ndb/ Accessed on April, 2018.

## Chemical Composition

### Nutritional Composition

Chinese kiwifruit, known as the “king of fruits,” is a fruit with high-pulp juices, thick flesh, delicious taste, and rich nutrition and has a higher commercial and economic value. It is a rich source of various nutrients including vitamins, carbohydrate, sugar, minerals, amino acids, protein, fatty acids (e.g., linoleic acid), and carotenoids. **Table 2** lists the nutritional composition of sun-gold kiwifruit reported from the USDA Food Composition Database (United States Department of Agriculture, USDA Food Composition Databases, 2018). **Table 3** shows the chemical content of *A. chinensis* fruit (Chang and Case, 2005; Cui et al., 2007; Zhou et al., 2009; Xu et al., 2010; He et al., 2014; He et al., 2015a; He et al., 2015b; Xu et al., 2016; Twidle et al., 2018; Sivakumaran et al., 2018; Wei et al., 2018; Zhang et al., 2018). Of particular note, nutritional composition in kiwifruit is vitamin C (1.61 mg/g) and minerals K (3.15 mg/g). The average vitamin C content of Huayou, Jintao, Ganmi-1, Ganmi-2, Ganmi-3, Wuzhi-3, and Cuiyu cultivated in China are 1.59, 1.49, 0.86, 1.34, 0.97, 2.88, and 1.18 mg/g, respectively. Meanwhile, the vitamin C in SunGold was 1.61 mg/g edible flesh, followed by other varieties Sweet Green (1.5 mg/g) and green “Hayward” (0.85 mg/g) (Sivakumaran et al., 2018). Especially, the vitamin C content in kiwifruit is higher than that determined in lemon, orange, strawberry, and grapefruits (Ma et al., 2017).

**Table 3:** The nutritional composition or phytochemicals content of *A. chinensis* fruit.

Composition	Cultivar location	Genotype	Method	Plant part	Content	Ref.
Vitamin C (ascorbic acid)	Colomicta	A. chinensis	HPLC	Ripe fruits	0.82 mg/g FW; 4.34 mg/g DW	Kvesitadze et al., 2001
Vitamin C	Cardinal	A. chinensis	HPLC	Ripe fruits	0.74 mg/g FW; 4.30 mg/g DW	Kvesitadze et al., 2001
Vitamin C	Bruno	A. chinensis	HPLC	Ripe fruits	0.76 mg/g FW; 4.28 mg/g DW	Kvesitadze et al., 2001
Vitamin C	Monti	A. chinensis	HPLC	Ripe fruits	0.76 mg/g FW; 4.33 mg/g DW	Kvesitadze et al., 2001
Vitamin C	Purpuria	A. chinensis	HPLC	Ripe fruits	0.78 mg/g FW; 4.27 mg/g DW	Kvesitadze et al., 2001
Vitamin C	Gaivard	A. chinensis	HPLC	Ripe fruits	0.72 mg/g FW; 4.14 mg/g DW	Kvesitadze et al., 2001
Vitamin C	Gaivard	A. chinensis	HPLC	Skin	0.21 mg/g FW; 0.63 mg/g DW	Kvesitadze et al., 2001
Vitamin C	Gaivard	A. chinensis	HPLC	Pulp	0.85 mg/g FW; 4.75 mg/g DW	Kvesitadze et al., 2001
Vitamin C	Gaivard	A. chinensis	HPLC	Core	0.48 mg/g FW; 2.67 mg/g DW	Kvesitadze et al., 2001
Vitamin C	Gaivard	A. chinensis	HPLC	Fresh juice	0.55 mg/g FW; 3.44 mg/g DW	Kvesitadze et al., 2001
Vitamin C	Gaivard	A. chinensis	HPLC	Juice after 24 h	0.55 mg/g FW; 3.44 mg/g DW	Kvesitadze et al., 2001
Vitamin C	Shaanxi, China	Huayou	2,6-dichloroindophenol titration method	Ripe fruits	1.59 mg/g FW	Ma et al., 2017
Total starch content in tissue	Pukekohe	Zespri^®^ SunGold Kiwifruit	Total starch assay kit	Outer pericarp	38.6% DW	Li and Zhu, 2017
Total starch content in tissue	Auckland	Gold9	Total starch assay kit	Outer pericarp	51.8% DW	Li and Zhu, 2017
Total starch content in tissue	New Zealand	Hort16A	Total starch assay kit	Outer pericarp	44.8% DW	Li and Zhu, 2017
Apparent amylose content	Pukekohe	Zespri^®^ SunGold Kiwifruit	Total starch assay kit	Outer pericarp	27.5% DW	Li and Zhu, 2017
Apparent amylose content	Auckland	Gold9	Total starch assay kit	Outer pericarp	24.5% DW	Li and Zhu, 2017
Apparent amylose content	New Zealand	Hort16A	Total starch assay kit	Outer pericarp	25.3% DW	Li and Zhu, 2017
True amylose content	Pukekohe	Zespri^®^ SunGold Kiwifruit	Total starch assay kit	Outer pericarp	17.8% DW	Li and Zhu, 2017
True amylose content	Auckland	Gold9	Total starch assay kit	Outer pericarp	15.7% DW	Li and Zhu, 2017
True amylose content	New Zealand	Hort16A	Total starch assay kit	Outer pericarp	15.5% DW	Li and Zhu, 2017
Total dietary fibre	New Zealand	Hort 16A	Megazyme method	puree	34.1 mg/g FW	Yuliarti et al., 2008
Total dietary fibre	New Zealand	Hort 16A	Megazyme method	Skin and cores	13.84% DW	Yuliarti et al., 2008
Insoluble dietary fibre	New Zealand	Hort 16A	Megazyme method	Puree	26.1 mg/g FW	Yuliarti et al., 2008
Insoluble dietary fibre	New Zealand	Hort 16A	Megazyme method	Skin and cores	11.39% DW	Yuliarti et al., 2008
Soluble dietary fibre	New Zealand	Hort 16A	Megazyme method	Puree	8 mg/g FW	Yuliarti et al., 2008
Soluble dietary fibre	New Zealand	Hort 16A	Megazyme method	Skin and cores	2.45% DW	Yuliarti et al., 2008
Nonstarch polysaccharide	New Zealand	gold kiwifruit	Acid extraction	Pomace	77.59% DW	Yuliarti et al., 2015a
Nonstarch polysaccharide	New Zealand	gold kiwifruit	Acid extraction	Early-harvested Fruits	69.14% DW	Yuliarti et al., 2015b
Nonstarch polysaccharide	New Zealand	gold kiwifruit	Acid extraction	Main-harvested fruits	64.49% DW	Yuliarti et al., 2015b
Nonstarch polysaccharide	New Zealand	gold kiwifruit	Water extraction	Pomace	79.16% DW	Yuliarti et al., 2015a
Nonstarch polysaccharide	New Zealand	gold kiwifruit	Water extraction	Early-harvested fruits	60.74% DW	Yuliarti et al., 2015b
Nonstarch polysaccharide	New Zealand	gold kiwifruit	Water extraction	Main-harvested fruits	63.77% DW	Yuliarti et al., 2015b
Nonstarch polysaccharide	New Zealand	gold kiwifruit	Enzymatic extraction	Pomace	80.12% DW	Yuliarti et al., 2015a
Nonstarch polysaccharide	New Zealand	gold kiwifruit	Enzymatic extraction	Early-harvested fruits	39.21% DW	Yuliarti et al., 2015b
Nonstarch polysaccharide	New Zealand	gold kiwifruit	Enzymatic extraction	Main-harvested fruits	64.02% DW	Yuliarti et al., 2015b
Total free amino acids	Shaanxi, China	Hort16A	Hitachi L-8900 amino acid analyzer	Ripe fruits	8.31 mg/g FW	Ma et al., 2017
Total free amino acids	New Zealand	Hort16A	Hitachi L-8900 amino acid analyzer	Ripe fruits	8.01 mg/g FW	Ma et al., 2017
Total free amino acids	Shaanxi, China	Huayou	Hitachi L-8900 amino acid analyzer	Ripe fruits	7.15 mg/g FW	Ma et al., 2017
Total essential amino acids	Shaanxi, China	Huayou	Hitachi L-8900 amino acid analyzer	Ripe fruits	1.55 mg/g FW	Ma et al., 2017
Total essential amino acids	Shaanxi, China	Hort16A	Hitachi L-8900 amino acid analyzer	Ripe fruits	2.09 mg/g FW	Ma et al., 2017
Total essential amino acids	New Zealand	Hort16A	Hitachi L-8900 amino acid analyzer	Ripe fruits	2.06 mg/g FW	Ma et al., 2017
Nonessential amino acids	New Zealand	Hort16A	Hitachi L-8900 amino acid analyzer	Ripe fruits	5.95 mg/g FW	Ma et al., 2017
Nonessential amino acids	Shaanxi, China	Hort16A	Hitachi L-8900 amino acid analyzer	Ripe fruits	6.22mg/g FW	Ma et al., 2017
Nonessential amino acids	Shaanxi, China	Huayou	Hitachi L-8900 amino acid analyzer	Ripe fruits	5.60 mg/g FW	Ma et al., 2017
Total phenolic	New Zealand	Zespri^®^ SunGold Kiwifruit	Folin-Ciocalteu method	Thinned young fruits (20 days)	∼80 mg GAE/g FDW	Jiao et al., 2019
Total phenolic	Shanxi Province	Red sun	Folin-Ciocalteu method	Ripe fruits	0.87 mg GAE/g FW	Wang et al., 2018
Total phenolic	Shanxi Province	Cuiyu	Folin-Ciocalteu method	Ripe fruits	0.83 mg GAE/g FW	Wang et al., 2018
Total flavonoid	New Zealand	Zespri^®^ SunGold Kiwifruit	UV/Vis	Thinned young fruits (20days)	∼30 mg CE/g FDW	Jiao et al., 2019
Total flavanol	New Zealand	Zespri^®^ SunGold Kiwifruit	UV/Vis	Thinned young fruits (20days)	∼20 mg CE/g FDW	Jiao et al., 2019
Total flavonoid	Shanxi Province	Red sun	UV/Vis	Ripe fruits	0.68 mg CE/g FW	Wang et al., 2018
Total flavonoid	Shanxi Province	Cuiyu	UV/Vis	Ripe fruits	0.68 mg CE/g FW	Wang et al., 2018
Total carotenoid	New Zealand	Hort16A	HPLC	Main-harvested fruits	0.62 mg/100 g FW	McGhie and Ainge, 2002
Total chlorophylls	New Zealand	gold kiwifruit	HPLC	Outer Pericarp	0.07 mg/100 g FW	Montefiori et al., 2005
Total anthocyanins	New Zealand	Hongyang	HPLC	Pericarp	2.99 mg/100 g FW	Montefiori et al., 2005
Total organic acids	China	Hongyang	HPLC	Ripe Fruits	39.86 mg/g FW	Montefiori et al., 2005
Total organic acids	China	Cuiyu	HPLC	Ripe Fruits	29.65 mg/g FW	Montefiori et al., 2005

The data show evidence that the sun-gold kiwifruit is high in carbohydrate (15.79%) and sugars (12.3%). The total starches contents were found for outer pericarp and core tissues ranged from 38.6% to 51.8% and 34.6% to 40.7% DW in three harvesting *A. chinensis* varieties, and the starches in core have higher amylose content (20.7%–23.3%) and enzyme susceptibility. However, the crystallinity degree, granule size, and gelatinization parameters of starches in core are somewhat lower (Li and Zhu, 2017). The kiwifruit peel contains a higher total pectin content (3.7%–4.2%) than that of pulp (1.6%–2.1%) (Meng et al., 2017). Xia et al. (2017) analyzed the polysaccharide from Hongyang using water extraction, followed by column chromatography, high performance gel permeation chromatography, HPLC, and Fourier transform infrared spectroscopy. The results indicated that the polysaccharide of Hogyang fruit consisted of the following monosaccharides: D-galactose (25.45%), D-galacturonic acid (25.25%), L-arabinose (20.51%), L-rhamnose (17.78%), D-glucose (6.14%), D-mannose (2.13%), D-xylose (1.03%), D-glucuronic acid (0.97%), and D-fucose (0.74%). These studies confirmed the utilization potential of Chinese kiwifruit as an incredibly healthy food and loaded with important nutrients and health benefits for human consumption.

Kiwifruit contained 18 free amino acids. Briefly, the total essential amino acid contents in Jintao, Hongyang, Huayou, and Hort16A cultivated in China are 2.59, 1.55, 2.0, and 2.09 mg/g FW, whereas the total essential amino acid in Hort16A cultivated in New Zealand was 2.06 mg/g FW. Meanwhile, Jintao, Hort16A and Hongyang also had a high amount of nonessential amino acid and total free amino acid. The most abundant amino acids detected in the kiwifruit were arginine, glutamic acid, lysine, phenylalanine, aspartic acid, and tyrosine (Ma et al., 2017). There are a number of total saturated lipids including C8:0, C10:0, C12:0, C14:0, C16:0, and C18:0 with the content of 1.49, 0.05, 0.14, 0.14, 0.09, 0.9, and 0.14 mg/g in edible flesh portion of *A. chinensis*. Meanwhile, the content of monounsaturated fatty acids C16:1 and C18:1 are 0.09 and 0.27 mg/g, and the polyunsaturated fatty acids C18:2 and C18:3 are 1.13 and 0.77 mg/g (Drummond, 2013). In fact, the kiwifruit seed oil is rich in unsaturated fatty acids (89.92%), notably linolenic acid, which accounts for 60.59% of total seed oil (Luan et al., 2017). Υ-tocopherol, Υ-tocotrienol, and ƍ-tocotrienol are identified in kiwifruit seed oil (Fiorentino et al., 2009). Besides, minerals like calcium, iron, potassium, magnesium, sodium, phosphorus, copper, manganese, zinc, iodine, selenium, and vitamins including vitamin A, β-carotene, lutein, zeaxanthin, riboflavin, niacin, pantothenic acid, vitamin B6, folate, tocopherol, vitamin E, vitamin K, and choline are identified in kiwifruit (Sivakumaran et al., 2018). Thus, these data suggest that kiwifruit is an interesting fruit for daily nutrition and energy suppliers.

### Phytochemicals

A range of phytochemicals, including triterpenoids, saponins, and phenolic compounds (flavonoids, polyphenols, anthraquinones, and coumarins) varying in structures, were found and identified in *A. chinensis*. The major constituents isolated and identified in leaves and roots of *A. chinensis* are listed in **Table 4**.

**Table 4:** Chemical constituents isolated from *A. chinensis*.

NO	Name	Cas	Formula	Source	Ref.
Triterpenoids					
1.	(2α,​3β,​4α)​-​2,​3,​23-​Trihydroxyursa-​12,​20(30)​-​dien-​28-​oic acid; Actinidic acid	341971-45-7	C_30_ H_46_ O_5_	roots, unripe fruit	Ji and Liang, 1985; Lahlou et al., 2001
2.	Maslinic acid	4373-41-5	C_30_ H_48_ O_4_	roots	Cui et al., 2007
3.	Ursolic acid acetate	7372-30-7	C_32_ H_50_ O_4_	roots	Cui et al., 2007
4.	23-​Hydroxyursolic acid	94414-19-4	C_30_ H_48_ O_4_	roots	Cui et al., 2007
5.	Ergosta-​4,​6,​8(14)​,​22-​tetraen-​3-​one	19254-69-4	C_28_ H_40_ O	roots	Cui et al., 2007
6.	2α,​3β,​24-​Trihydroxyurs-​12-​en-​28-​oic acid	143839-02-5	C_30_ H_48_ O_5_	roots	Ji and Liang, 1985
7.	2α,​3α,​24-​Trihydroxyurs-​12,​20(30)​-​dien-​28-​oic acid	341503-22-8	C_30_ H_46_ O_5_	roots	Ji and Liang, 1985
8.	Pygenic acid A (3- *epi*-corosolic acid)	52213-27-1	C_30_ H_48_ O_4_	roots	Chen et al., 2011
9.	2α,3β-Dihydroxyurs-12-en-28,30-olide	1198363-27-7	C_30_ H_46_ O_4_	roots	Zhou et al., 2009
10.	2α,3β,24-Trihydroxyurs-12-en-28,30-olide	1198363-28-8	C_30_ H_46_ O_5_	roots	Zhou et al., 2009
11.	3β-Hydroxyurs-12,18-dien-28-oic acid	14021-14-8	C_30_ H_46_ O_3_	roots	Zhou et al., 2009
12.	2α,3α,23-Trihydroxyursa-12, 20(30)-dien-28-oic acid	1187824-97-0	C_30_ H_46_ O_5_	roots	Zhou et al., 2009
13.	2α,3α,19α,23, 24-Pentahydroxyurs-12-en-28-oic acid	1309360-33-5	C_30_ H_48_ O_7_	roots	Xu et al., 2010
14.	Ursolic acid	74984-66-0	C_30_ H_48_ O_3_	roots	Xu et al., 2010
15.	Pseudotaraxasterol	464-98-2	C_30_ H_50_ O	roots	Xu et al., 2010
16.	2α,3α,23-Trihydroxyurs-12-en-28-oic acid	103974-74-9	C_30_ H_48_ O_5_	roots	Xu et al., 2010
17.	2α,3β,24-Trihydroxyurs-12-en-28-oic acid	475631-15-3	C_30_ H_48_ O_5_	roots	Xu et al., 2010
18.	2α,3β,19α, 23-Tetrahydroxyurs-12-en-28-oic acid	70868-78-9	C_30_ H_48_ O_6_	roots	Xu et al., 2010
19.	2α,3α,19α, 24- Tetrahydroxyurs-12-en-28-oic acid 28-O-β-D-glucopyranoside	153753-66-3	C_36_ H_58_ O_11_	roots	Xu et al., 2010
20.	Oleanolic acid acetate	4339-72-4	C_32_ H_50_ O_4_	roots	Zhu et al., 2013
21.	Corosolic acid	4547-24-4	C_30_ H_48_ O_4_	roots	Zhu et al., 2013
22.	Arjunic acid	31298-06-3	C_30_ H_48_ O_5_	roots	Zhu et al., 2013
23.	Euscaphic acid	53155-25-2	C_30_ H_48_ O_5_	roots	Zhu et al., 2013
24.	Oleanolic acid	508-02-1	C_30_ H_48_ O_3_	roots	He et al., 2014
25.	2α,​3α,​24-​Trihydroxyolean-​12-​en-​28-​oic acid	150821-16-2	C_30_ H_48_ O_5_	roots	He et al., 2015a
26.	2α,​3α,​19α,​24-​Tetrahydroxyurs-​12-​en-​28-​oic acid	153753-65-2	C_30_ H_48_ O_6_	roots	He et al., 2015a
27.	Jacoumaric acid	63303-42-4	C_39_ H_54_ O_6_	roots	Cui, 2016
28.	3β-​Hydroxystigmast-​5-​en-​7-​one	2034-74-4	C_29_ H_48_ O_2_	roots	Xu et al., 2016
29.	(2α,​3α)​-​2,​3,​23,​24-​Tetrahydroxyurs-​12-​en-​28-​oic acid; 2α,​3α,​23,​24-​Tetrahydroxy ursan-​12-​en-​28-​acid	143773-49-3	C_30_ H_48_ O_6_	roots	Xu et al., 2016
30.	Oleanan-​28-​oic acid, 12-​chloro-​2,​3,​13,​23-​tetrahydroxy-​, γ-​lactone, (2α,​3β,​4α,​12α)​-	1309360-32-4	C_30_ H_47_ Cl O_5_	roots	Xu et al., 2016
31.	Urs-​13(18)​-​en-​28-​oic acid, 2,​3,​23-​trihydroxy-​, (2α,​3β,​4α)​-	1980812-62-1	C_30_ H_48_ O_5_	roots	Xu et al., 2016
32.	Urs-​13(18)​-​en-​28-​oic acid, 2,​3,​19,​23-​tetrahydroxy-​, β-​D-​glucopyranosyl ester, (2α,​3β,​4α)​-	1980812-63-2	C_36_ H_58_ O_11_	roots	Xu et al., 2016
33.	Pygenic acid B (2α,3α,24-trihydroxyurs-12-en-28-oic acid)	89786-83-4	C_30_ H_48_ O_5_	roots	Xu et al., 2016
34.	2α,3α,23,24-Tetrahydroxyursa-12, 20(30)-dien-28-oic acid	2220160-45-0	C_30_ H_46_ O_6_	roots	Wei et al., 2018
35.	2α,3β,23,24-Tetrahydroxyurs-12-en-28-oic acid	116787-94-1	C_30_ H_48_ O_6_	roots	Wei et al., 2018
36.	2α,3β,23-Trihydroxyurs-12-en-28-oic acid	114580-55-1	C_30_ H_48_ O_5_	roots	Wei et al., 2018
37.	3β-Hydroxyurs-12-en-28-oic acid	77-52-1	C_30_ H_48_ O_3_	roots	Wei et al., 2018
38.	3β-Hydroxyolean-12-en-28-oic acid	28283-45-6	C_35_ H_56_ O_7_	roots	Wei et al., 2018
39.	2β,3α,23-Trihydroxyurs-12-en-28-oic acid	175132-32-8	C_30_ H_48_ O_5_	roots	Wei et al., 2018
40.	2β,3β,23-Trihydroxyurs-12-en-28-oic acid	116348-15-3	C_3o_ H4_8_ O_5_	roots	Wei et al., 2018
41.	Spathodic acid 28-O-β-glucopyranoside	870559-41-4	C_36_ H_58_ O_10_	root barks	Zhang et al., 2018
42.	Fupenzic acid	119725-20-1	C_3o_ H_44_ O_5_	root barks	Zhang et al., 2018
Phenols					
43.	Planchol A	883238-17-3	C_14_ H_14_ O_6_	roots	Chang and Case, 2005
44.	Planchol B	883238-19-5	C_15_ H_16_ O_6_	roots	Chang and Case, 2005
45.	Planchol C	883238-20-8	C_16_ H_18_ O_6_	roots	Chang and Case, 2005
46.	Planchol D	883238-21-9	C_16_ H_16_ O_7_	roots	Chang and Case, 2005
47.	Benzeneacetic acid, 2-​[(3,​4-​dihydroxybenzoyl)​oxy]​-​4,​6-​dihydroxy-​, methyl ester	911315-93-0	C_16_ H_14_ O_8_	leaves	Wurms and Cooney, 2006
48.	Tachioside (methoxyhydroquinone-3-O-β-D-glucopyranoside)	109194-60-7	C_13_ H_18_ O_8_	roots	Zhou et al., 2010
49.	Isotachioside (methoxyhydroquinone-1-O-β-D-glucopyranoside)	31427-08-4	C_13_ H_18_ O_8_	roots	Zhou et al., 2010
50.	Vanillic acid	121-34-6	C_8_ H_8_ O_4_	roots	Zhou et al., 2010
51.	1-O-(β-D-glucosyl)-2-[2-methoxy-4-(ω-hydroxypropyl)-phenoxy]-propan-3-ol	68340-35-2	C_19_ H_30_ O_10_	roots	Zhou et al., 2010
52.	Protocatechualdehyde	139-85-5	C_7_ H_6_ O_3_	roots	He et al., 2014
53.	rel-​(1R,​2R)​-​1,​2-​Bis(4-​hydroxy-​3-​methoxyphenyl)​-​1,​3-​propanediol	69887-40-7	C_17_ H_20_ O_6_	roots	He et al., 2014
54.	rel-​(1R,​2S)​-​1,​2-​Bis(4-​hydroxy-​3-​methoxyphenyl)​-​1,​3-​propanediol	69887-41-8	C_17_ H_20_ O_6_	roots	He et al., 2014
55.	p-​Hydroxyl benzoic acid	99-96-7	C_7_ H_6_ O_3_	roots	He et al., 2014
56.	Chlorogenic acid	327-97-9	C_16_ H_18_ O_9_	roots	He et al., 2015a
57.	Caffeic acid	331-39-5	C_9_ H_8_ O_4_	roots	He et al., 2015a
58.	Cryptochlorogenic acid	905-99-7	C_16_ H_18_ O_9_	roots	He et al., 2015a
59.	Neochlorogenic acid	906-33-2	C_16_ H_18_ O_9_	roots	He et al., 2015a
60.	5-​O-​Coumaroylquinic acid	87099-71-6	C_16_ H_18_ O_8_	roots	He et al., 2015a
61.	Dihydroxy-​dihydrochalcone-​2’-​O-​β-​D-​glucopyranoside	23140-78-5	C_21_ H_24_ O_9_	roots	Xu et al., 2016
Flavonoids					
62.	Epicatechin	490-46-0	C_15_ H_14_ O_6_	unknown	Michaud and Ane-Margail, 1977
63.	epi-​Afzelechin	24808-04-6	C_15_ H_14_ O_5_	unknown	Michaud and Ane-Margail, 1977
64.	Procyanidin C_1_	37064-30-5	C_45_ H_38_ O_18_	unknown	Michaud and Ane-Margail, 1977
65.	2-​(3,​4-​Dihydroxyphenyl)​-​3,​4-​dihydro-​4-​[(phenylmethyl)​thio]​-​2H-​1-​benzopyran-​3,​5,​7-​triol	66052-27-5	C_22_ H_20_ O_6_ S	unknown	Michaud and Ane-Margail, 1977
66.	2,​2’-​Bis(3,​4-​dihydroxyphenyl)​-​3,​3’,​4,​4’-​tetrahydro-​4’-​[(phenylmethyl)​thio]​[4,​8’-​bi-​2H-​1-​benzopyran]​-​3,​3’,​5,​5’,​7,​7’-​hexol	66293-44-5	C_37_ H_32_ O_12_ S	unknown	Michaud and Ane-Margail, 1977
67.	Afzelechin	2545-00-8	C_15_ H_14_ O_5_	roots	Chang and Case, 2005
68.	Procyanidin B_3_	23567-23-9	C_30_ H_26_ O_12_	roots	Chang and Case, 2005
69.	Procyanidol B_2_	29106-49-8	C_30_ H_26_ O_12_	roots	Chang and Case, 2005
70.	Afzelechin-​(4α→8)​-​afzelchin	101339-37-1	C_30_ H_26_ O_10_	roots	Chang and Case, 2005
71.	(2R,​2’R,​3R,​3’*R*,​4*R*)​-​3,​3’,​4,​4’-​Tetrahydro-​2,​2’-​bis(4-​hydroxyphenyl)​[4,​8’-​bi-​2*H*-​1-​benzopyran]​-​3,​3’,​5,​5’,​7,​7’-​hexol	114715-48-9	C_30_ H_26_ O_10_	roots	Chang and Case, 2005
72.	Quercetin	117-39-5	C_15_ H_10_ O_7_	fruits	Lee et al., 2010
73.	(+)-Catechin	154-23-4	C_15_H_14_O_6_	roots	Zhou et al., 2010
74.	(-​)​-​Epicatechin-​5-​*O*-​β-​D-​glucopyranoside	131831-20-4	C_21_ H_24_ O_11_	roots	He et al., 2014
Anthraquinones					
75.	Emodic acid	478-45-5	C_15_ H_8_ O_7_	roots	Ji and Liang, 1985
76.	Hydroxyemodin	481-73-2	C_15_ H_10_ O_6_	roots	Ji and Liang, 1985
77.	Emodin	518-82-1	C_15_ H_10_ O_5_	roots	Ji and Liang, 1985
78.	Emodin 3-​methyl ether	521-61-9	C_16_ H_12_ O_5_	roots	Ji and Liang, 1985
79.	Questin	3774-64-9	C_16_ H_12_ O_5_	roots	Ji and Liang, 1985
Coumarins					
80.	5-Hydroxy-6-methoxy-7-O-β-D-glucosyl coumarin	141238-32-6	C_16_ H_18_ O_10_	roots	Zhou et al., 2010
81.	Fraxin	524-30-1	C_16_ H_18_ O_10_	roots	Zhou et al., 2010
82.	Esculin	531-75-9	C_15_ H_16_ O_9_	roots	He et al., 2015b
83.	Isofraxoside	24778-11-8	C_16_ H_18_ O_10_	roots	He et al., 2015b
Other compouds					
84.	β-​Sitosterol	83-46-5	C_29_ H_50_ O	roots	Ji and Liang, 1985
85.	Butyl β-D-fructopyranoside	67884-27-9	C_10_ H_20_ O_6_	roots	Zhou et al., 2010
86.	Lignoceric acid	557-59-5	C_24_ H_48_ O_2_	roots	Chen et al., 2011
87.	(-​)​-​Quinic acid γ-​lactone	665-27-0	C_7_ H_10_ O_5_	roots	Chen et al., 2011
88.	Stearyl-β-D-glucopyranoside	76739-16-7	C_24_ H_48_ O_6_	roots	Chen et al., 2011
89.	Daucosterol	474-58-8	C_35_ H_60_ O_6_	roots	Chen et al., 2011
90.	Indole-​3-​carboxylic acid	771-50-6	C_9_ H_7_ N O_2_	roots	He et al., 2014
91.	Stigmastane-3,6-diol	112244-29-8	C_29_ H_52_ O_2_	roots	Cui, 2016
92.	Sitoindoside Ⅰ	18749-71-8	C_51_ H_90_ O_7_	roots	Cui, 2016

### Triterpenoids

Currently, triterpenoids have been the major research focus of *A. chinensis* components due to their promising antitumor properties. To date, 42 triterpenoids have been isolated and identified mainly from roots of *A. chinensis*. The commonly triterpenoids found in roots of *A. chinensis* are 12-en-28-oic acids of oleanane and ursane type. It is noteworthy that some of these triterpenoids (1-2, 7, 15-18, 21, 25-26, 29-30, and 34-40) have significant antitumor activity and deserve further research and development.

### Phenolic Compounds

The phenolic compounds abundantly presented in different botanical parts of *A. chinensis*, and they have drawn increasing attention. These compounds include phenols, flavonoids, and flavanols are characterized by antitumor, antioxidant, and free radicals scavenging properties. HPLC-PAD and UPLC-QqQ-MS/MS-based methods have been used generally for the identification and quantification of these phenolic compounds (Ma et al., 2017; Jiao et al., 2019). The total phenolic, flavonoid, and flavanol contents from young *A. chinensis* kiwifruits “Zespri^®^ SunGold Kiwifruit” growing in 20 days are 82.84 mg GAE/g FDW, 30.08 catechin/g equivalents FDW, and 20.20 catechin/g equivalents FDW. Meanwhile, the total phenolic, flavonoid, and flavanol contents presented in young *A. chinensis* kiwifruits growing in 60 days and mature kiwifruits are gradually decreasing, indicating polyphenol content possesses a decreasing pattern during fruit ripening (Jiao et al., 2019). The major chemical composition of phenolics detected in young “Zespri^®^ SunGold Kiwifruit” are epicatechin, quercitrin, rutin, catechin, chlorogenic acid, ferulic acid, and vanillic acid. Based on UPLC-TOF/MS and UPLC-QqQ/MS method, Zhao et al., 2014 showed that the radix *A. chinensis* contained catechin derivatives, quinic acid derivatives, coumarin derivatives, caffeic acid, and *p*-coumaric acid (Zhao et al., 2014), showing that *A. chinensis* appears to be a good source of phenolics.

### Volatile Compound and Essential Oil

The volatile components of *A. chinensis* var. *chinensis* fruit and flowers have been profiled by GC-MS. The dominant volatile components of eating-ripe firmness fruit are straight-chain aldehydes, alcohols, and esters, such as hexanal, decanal, octanal, nonanal, benzaldehyde, acetaldehyde, hex-E2-enal, 1,8-cineole, ethanol, hexanol, methyl butanoate, and ethyl octanoate (Wang et al., 2011). The volatile components of flowers included (3E,6E)-α-farnesene (38.8%), pentadecane (12.49%), (+)-germacrene D (8.55%), heptadecane (8.01%), (8Z)-heptadecene (7.72%), 2-phenylethano (4.69%), (3Z,6Z,9Z)-heptadecatriene (2.54%), and nonadecane (1.98%) (Twidle et al., 2018). It can be found that terpenes and straight chain alkenes were dominant in flowers of *A. chinensis* var. *chinensis*, which contained nearly >92% of the total ion counts. Importantly, many of these compounds possess strong and interesting aroma. However, the volatile components gradually changed during maturation. The essential oil of roots of *A. chinensis* have been profiled by GC-MS, and the major essential oil in roots are dodecane (29.39%), octane (5.16%), decane (2.94%), paeonal (2.81%), camphor (2.77%), *n*-decanoic acid (2.64%), 4-Methyldodecane (2.45%), undecane (2.16%), and linalool oxide (2.1%) (Yu et al., 2009).

### Carotenoid and Chlorophyll

Carotenoids and chlorophyll are responsible for the color and attractiveness of kiwifruit fruits, as well as provide nutritional values. The carotenoids detected in the red-fleshed genotypes of *A. chinensis* fruit (Hort16A) are 9′-cis-neoxanthin, violaxanthin, antheraxanthin, lutein, zeaxanthin, β-cryptoxanthin, and β-carotene (McGhie and Ainge, 2002; Montefiori et al., 2005). Chlorophylls a and b are the dominant chlorophylls in Hort16A (McGhie and Ainge, 2002).

### Quality Determination

Ripe kiwifruit is susceptible to environmental and itself. Usually, human sensory evaluation method can directly identify the fruit shape, color, surface, pulp, and flavor, but there is little information about swelling, ripening, and other agents present in fruit. Physical and chemical method including firmness and microbial are used effectively to determine the quality condition of kiwifruit. Some new instrument detection methods with accurate analysis ability such as GC, GC-MS, HPLC, UPLC-QqQ-MS/MS, and electronic nose combined with surface acoustic wave resonator are developed for the fruit and its products quality rapid analysis (Kvesitadze et al., 2001; Montefiori et al., 2005; Liu and Hui, 2015; Jiao et al., 2019). As to radix *A. chinensis*, the systematical method like UPLC-TOF/MS and UPLC-QqQ/MS is commonly applied to quality evaluation and active components analysis for *A. chinensis* (Zhao et al., 2014). Therefore, there are many high accurate analysis methods for rapid quality evaluation, but there is a lack of effective and standardized quality and safety standard for kiwifruit in China. Thus, there is an urgent demand for developing specific functional components and quality evaluation indicators for standardization and quality control of the fruit and its products.

### Biological Activities

*A. chinensis* contains a range of bioactive compounds accounting for natural pharmacological properties including antitumor, antioxidant, anti-inflammatory, immunoregulatory, hypolipemic, antidiabetic, and cardiovascular protective activities, and most of these biological activities support its traditional use. **Table 5** shows the major biological activities of compound or extract from *A. chinensis*.

**Table 5:** Biological activities of compounds or extracts of *A. chinensis*.

Effect	Compound/Extract	Class of compounds	In vitro	In vivo	Ref.
a	1	A	Showed cytotoxicities against HepG2, A549, MCF-7, and SK-OV-3 with IC_50_ (48 h) values of 36.4, 40.37, 44. 3, and 16.33 μM.		Wei et al., 2018
	2	A	Showed cytotoxicities against A549, LoVo, and HepG2 with IC_50_ (48 h) values of 23.2, 6, and 34.9 μg/ml.		Wei et al., 2018; Xu et al., 2010
	7	A	Showed cytotoxicities against A549, MCF-7, SK-OV-3, and HeLa with IC_50_ (48 h) values of 16.63, 47.93, 22.91, and 15.27 μM.		Wei et al., 2018
	15	A	Showed cytotoxicities against LoVo, and HepG2 with IC_50_ (48 h) values of 31.1, and 33.9 μg/ml.		Xu et al., 2010
	16	A	Showed cytotoxicities against HepG2, MCF-7, SK-OV-3, and HeLa with IC_50_ (48 h) values of 12.22, 36.29, 45.13, and 49.71 μM.		Wei et al., 2018
	17	A	Showed cytotoxicities against A549, MCF-7, SK-OV-3, and HeLa with IC_50_ (48 h) values of 39.3, 11.01, 40.9 and 41.6 μM.		Wei et al., 2018
	18	A	Showed cytotoxicities against HepG2, A549, MCF-7, and HeLa with IC_50_ (48 h) values of 19.08, 32.08, 35.74, and 15.05 μM.		Wei et al., 2018
	21	A	Inhibited HCC cells migration by targeting the VEGFR2/Src/FAK pathway.		Ku et al., 2015
	21	A	Showed cytotoxicities against A549, LoVo, and HepG2 with IC_50_ (48 h) values of 34.6, 2.9, and 9.2 μg/ml.		Xu et al., 2010
	25	A	Showed cytotoxicities against A549 and SK-OV-3 with IC_50_ (48 h) values of 42.74 and 25.83 μM.		Wei et al., 2018
	26	A	Showed cytotoxicities against A549 and HeLa with IC_50_ (48 h) values of 22.6 and 29.35 μM.		Wei et al., 2018
	29	A	Showed cytotoxicities against A549 and SK-OV-3 with IC_50_ (48 h) values of 31.3 and 37.9 μM.		Wei et al., 2018
	30	A	Showed cytotoxicities against A549, LoVo, and HepG2 with IC_50_ (48 h) values of 30.4, 31.1, and 25.5 μg/ml.		Xu et al., 2010
	34	A	Showed cytotoxicities against HepG2, A549, MCF-7, and HeLa with IC_50_ (48 h) values of 19.62, 18.86, 45.94 and 28.74 μM.		Wei et al., 2018
	35	A	Showed cytotoxicities against HepG2, MCF-7, and SK-OV-3 with IC_50_ (48 h) values of 11.76, 12, and 10.3 μM.		Wei et al., 2018
	36	A	Showed cytotoxicities against HepG2, MCF-7, and SK-OV-3 with IC_50_ (48 h) values of 14.22, 16.99, 28.9 μM.		Wei et al., 2018
	37	A	Showed cytotoxicities against HepG2, A549, MCF-7, and SK-OV-3 with IC_50_ (48 h) values of 48.4, 12.7, 11.2, and 31.7 μM.		Wei et al., 2018
	38	A	Showed cytotoxicities against A549, MCF-7, and SK-OV-3 with IC_50_ (48 h) values of 34.45, 42.2 and 49.55 μM.		Wei et al., 2018
	39	A	Showed cytotoxicities against HepG2 with IC_50_ (48 h) values of 32.5 μM.		Wei et al., 2018
	40	A	Inhibited NCI-H460 cell proliferation by decreasing NF-κB expression. Showed cytotoxicities against SK-OV-3 with IC_50_ of 37.21 μM.		Cheng et al., 2015; Wei et al., 2018
	43	B	Showed cytotoxic activity against P-388 and A-549 cell lines with IC_50_ of 2.5 and 1.42 μM.		Chang and Case, 2005
	44	B	Showed cytotoxic activity against P-388 and A-549 cell lines with IC_50_ of 3.85 and 2.88 μM.		Chang and Case, 2005
	45	B	Showed cytotoxic activity against P-388 and A-549 cell lines with IC_50_ of 5.02 and 4.5 μM.		Chang and Case, 2005
	46	B	Showed cytotoxic activity against P-388 and A-549 cell lines with IC_50_ of 3.52 and 2.6 μM.		Chang and Case, 2005
b	vitamin E (ƍ-Tocomonoenol)	C	Radical-scavenging capacities on DPPH and O_2_ were 23.96 and 29.20%; hydroperoxide conjugate dienes formation and TBARS were 26.88 and 46.70%.		Fiorentino et al., 2009
	vitamin E (α-tocopherol)	C	Radical-scavenging capacities on DPPH and O_2_ were 25.21 and 27.07%. hydroperoxide conjugate dienes formation and TBARS were 33.08 and 53.01%.		Fiorentino et al., 2009
	vitamin E (ƍ-tocopherol)	C	Radical-scavenging capacities on DPPH and O_2_ were 23.4 and 29.273%; hydroperoxide conjugate dienes formation and TBARS were 25.48 and 43.2%.		Fiorentino et al., 2009
	polymeric proanthocyanidins fractionated by methanol- water (80:20, v/v)	D	IC_50_ for DPPH, ABTS were 105.3 and 74.7μg/ml; FRAP values is 7.4 mM VCE/g.		Chai et al., 2014
	polymeric proanthocyanidins fractionated by acetone-methanol-water (40:40:20, v/v/v)	D	IC_50_ for DPPH, ABTS were 67.7 and 60.1 μg/ml; FRAP values is 9.6 mM VCE/g.		Chai et al., 2014
	polymeric proanthocyanidins fractionated by acetone-water (70:30, v/v)	D	IC_50_ for DPPH, ABTS were 69.3 and 39.5 μg/ml; FRAP values is 9.6 mmol VCE/g.		Chai et al., 2014
	polyphenols compounds (55.10 mg GAE/g DW), contain *p*-hydroxybenzoic acid, protocatechuic acid, and *p*-coumaric acid.	B	10-50 µg/ml showed DPPH free radical scavenging.		Deng et al., 2016
	seed oil rich in unsaturated fatty acid from Hongyang	E	IC_50_ for DPPH, HO·scavenging capacity were 31.4 and 1.09; FRAP and ORAC values were 107.3 mg and 1.09 Trolox/kg.		Deng et al., 2018
	seed oil rich in unsaturated fatty acid from Huayou	E	IC_50_ for DPPH, HO·scavenging capacity were 33.7 and 1.12; FRAP and ORAC values were 72.0 mg and 1.72 Trolox/kg.		Deng et al., 2018
	seed oil rich in unsaturated fatty acid from Hort 16A	E	IC_50_ for DPPH, HO·scavenging capacity were 32.4 and 1.04; FRAP and ORAC values were 3.3 mg and 1.69 Trolox/kg.		Deng et al., 2018
	water-soluble polysaccharides	F	0.5-3 mg/ml showed DPPH radical scavenging activity, protection of the HEK 293 cells from H_2_O_2_ damage.		Zhang et al., 2015
c	polymeric proanthocyanidins fractionated by methanol- water (80:20, v/v)	D	Inhibited monophenolase and diphenolase activity with IC_50_ of 180.2 and 390.2 μg/ml.		Chai et al., 2014
	polymeric proanthocyanidins fractionated by acetone-methanol- water (40:40:20, v/v/v)	D	Inhibited monophenolase activity with IC_50_ of 80.1 and 192.6 μg/ml.		Chai et al., 2014
	polymeric proanthocyanidins fractionated by acetone-water (70:30, v/v)	D	Inhibited monophenolase activity with IC_50_ of 48.9 and 64.9 μg/ml.		Chai et al., 2014
d	polyphenols compounds (55.10 mg GAE/g DW), contain *p*-hydroxybenzoic acid, protocatechuic acid, *p*-coumaric acid, etc.	B	20, 40, 60 µg/ml for 12 h inhibit IL-1β and TNF-α secretion in LPS-induced RAW 264.7 cells.		Deng et al., 2016
	seed oil rich in fatty acids	E		1.0 and 3.0 ml/kg/day for 84 days down-regulated TNF-α, IL-6, IL-1β, COX-2 and iNOS in high-fat diet induced mice.	Qu et al., 2019
	water-soluble polysaccharides	F	50, 100, 200, 300 μg/ml reduce NO production of RAW 264.7 cells, and 100, 200 and 300 μg/ml enhanced phagocytic activity of RAW 264.7 cells.		Zhang et al., 2015
e	seed oil rich in fatty acids	E		1.0 and 3.0 mL/kg/day for 84 days decreased bodyweight and ameliorated serum TC, TG, HDL-C, and LDL-C levels in high-fat diet treated mice.	Qu et al., 2019
f	flavonoid-rich extract	G	IC_50_ of ACE inhibitory activity was 12.81 mg/ml.		Hettihewa et al., 2018
g	actinidin	H	Enhanced gastric protein α-, β-, and κ-caseins digestion under simulated gastric conditions.		Kaur et al., 2010
h	thaumatin-like protein	H	Inhibited *Botrytis cinereal*, *Mycosphaerella arachidicola* and *Coprinus comatus*, inhibit HIV-1 reverse transcriptase.		Wang and Ng, 2002
i	41	A	100 μg/ml inhibited tobacco mosaic virus with inhibition rate of 45.70%.		Zhang et al., 2018
j	21	A	50 μg/ml showed inhibitory effects on CYP2C19, CYP2D6, and CYP3A4 with 69.3,71.0 and 39.3 of remaining activity.		Xu et al., 2016
	25	A	10 μg/ml showed inhibitory effects on CYP2C9, CYP2C19, CYP2D6, and CYP3A4 with 28.3, 59.9, 31.8, and 37.1% of remaining activity.		Xu et al., 2016
	30	A	10 μg/ml showed inhibitory effects on CYP2C9 and CYP3A4 with 67.1 and 9.8% of remaining activity.		Xu et al., 2016
	33	A	50 μg/ml showed inhibitory effects on CYP2C19 and CYP3A4 with 75.0 and 35.0 of remaining activity		Xu et al., 2016
	61	B	10 μg/ml showed inhibitory effects on CYP2C9 with 69.0% of remaining activity.		Xu et al., 2016

a, Antitumor effects; b, Antioxidant activity; c. Antityrosinase activity; d, Anti-inflammatory activity; e, Hypolipidemic activity; f, ACE inhibitory activity; g, Digestive activity; h, Antifungal activity; i, Antiviral activity; j, Cytochrome P450 enzyme inhibitory activity. A, Triterpenoid; B, Phenols; C, Vitamin; D, Proanthocyanidins; E, Oil; F, Polysaccharides; G, Flavonoids; H, Protein.

### Antitumor Activity

Crude extracts, fractions, and isolated compounds from *A. chinensis* exhibited strong inhibition against tumor growth in various forms of human cancer cells. These cancer cells were hepatocellular carcinoma cells HepG2 (Xu et al., 2010; Zuo et al., 2012), Hep3B, SMMC7721, MHCC97L, MHCC97H, HCCLM3 (Fang et al., 2019), HL-7702 (He et al., 2017), Huh7 (Hou et al., 2018), lung cancer cells NCI-H460 and NCI-H1299 (Lv et al., 2018), colon cancer cells HT-29, LoVo, and SW480, pharyngeal carcinoma cell lines Fadu and HEP-2, gastric cancer cells SGC-7901, BGC-823, MKN-49P, and MFC, as well as other cancer cells like A549, P-388, MCF-7, SK-OV-3, and HeLa (Chang and Case, 2005; Xu et al., 2010; Xu et al., 2010; Zuo et al., 2012; Shen et al., 2014; Xia et al., 2017; Gu et al., 2017; Wang et al., 2017; Wei et al., 2018). These reported antitumor activities are consistent with the traditional usage such as liver cancer, lung cancer, colon cancer, esophagus cancer, and gastric cancer.

A large number of triterpenoids in roots of *A. chinensis* especially those with carboxyl group showed marked cytotoxicity against various types of cancer cells *in vitro*. Especially, compounds 1-2, 7, 15-18, 21, 25-26, 29-30, 34-40, and 43-46 exhibited remarkable antitumor activity against on A549, HepG2, LVOV, MCF-7, HeLa, and/or HepG2 *in vitro* (**Table 5**). Additionally, the polysaccharide of Hogyang fruit showed notable inhibitory against tumor cells lines SGC7901, MCF-7, HT29, HepG2, and NCI-H460 with IC_50_ of were 0.28, 0.31, 0.58, 0.64, and 0.65 µM, respectively (Xia et al., 2017). *In vivo*, a polysaccharide isolated from the roots of *A. chinensis* showed antitumor activity by prolonging the life of EAC or P388 cells-induced tumor mice and inhibiting the DNA synthesis in EAC cells (Lin, 1988). Early treatment and long-term treatment with water extracts of roots from *A. chinensis* with 2 g/kg/day strongly attenuated the malignant behavior of HCC in mice by decreasing DLX2 expression (Fang et al., 2019).

The molecular mechanism of the inhibition against tumor growth and the apoptosis promoting of the fractions and isolated compounds were due to downregulate *DLX2* gene expression and VEGFR2/Src/FAK pathway, inhibit cholesterol metabolism by upregulating PCSK9 signaling pathway, regulate gene encoding laminin subunit beta-3 pathways, and decreased NF-κB and EP3 expression. Meanwhile, the antioxidation and anti-inflammation are also important and possible mechanisms. The triterpenoids, polysaccharides, and phenolic compounds were identified as the major bioactive compounds in the extract from *A. chinensis* roots with antitumor properties (Chang and Case, 2005; Wei et al., 2018), which provides new way to search for treating cancers with natural therapeutic compounds. Overall, *A. chinensis* has prominent antitumor potential and has a good health benefit for people, however, the further *in vivo* and clinical studies on antitumor properties of *A. chinensis* are needed for confirmation.

### Antioxidant Activity

Antioxidant activity of bioactive compounds of *A. chinensis* have been the mostly studied by various *in vitro* and *in vivo* assays. These *in vitro* assays consisted of both chemical and biological assays like DPPH, ABTS, FRAP, HO·, ORAC, oxidative stress by H_2_O_2_, and lipid oxidation (Chai et al., 2014; Lee et al., 2015; Hwang et al., 2017; Deng et al., 2018). The *in vivo* assays were based on SOD, GSH, ALT, AST, oxidative DNA damage, and lipid oxidation (Iwasawa et al., 2011; Sun et al., 2017; Deng et al., 2018; Wang et al., 2018). The above results showed that *A. chinensis* is a good source of bioactive compounds with antioxidant properties to various extents. The antioxidant capacities of kiwifruit are greatly attributed to polyphenols, flavonoid, unsaturated fatty acid, and vitamin C. In addition, the different extraction methods, different plant parts, and genetic diversity of kiwifruit demonstrated different antioxidant activities. The peel showed the strongest antioxidant activity, followed by the pulp and the core. The antioxidant activity of kiwifruit peel was mainly depended on plenty of phenolic substances, and the antioxidant activity of the pulp was mainly attributed to the existence of a large amount of vitamin C (Zhang et al., 2016). The seed oil of Hort 16A and Hongyang are attractive materials rich in unsaturated fatty acid demonstrated radical scavenging capacities for FRAP, DPPH, HO·, and ORAC with IC_50_ of 3.3 mgTrolox/kg, 32.4 mg/ml, 1.04 mg/ml, 1.69 mgTrolox/kg, and 107.3 mgTrolox/kg, 31.4 mg/ml, 1.09 mg/ml, 1.99 mgTrolox/kg, respectively (Deng et al., 2018). The radical scavenging capacities of fresh and freeze-dried Hort 16A rich in phenolics and flavonoids for ABTS, DPPH, and ORAC were 8.8, 8.8, 98.3, and 6.0, 5.0, and 40.3 mg VCE/g, respectively (Hwang et al., 2017). The radical scavenging capacities of Red sun and Cuiyu rich in phenolics and flavonoids for ABTS, DPPH, ORAC, and FRAP were 1.35, 1.01, 10.78, 1.50 and 1.32, 0.9, 8.87, 1.28 mg VCE/g, respectively (Wang et al., 2018). Oral administration of kiwifruit protected lymphocytes against oxidative DNA damage, inhibit lipid oxidation in mice, increased SOD and GSH, and lowered ALT and AST levels in the patients (Sun et al., 2017). Therefore, *A. chinensis* possess confirmed antioxidant capacity and it seems that appropriate extraction methods, appropriate genotypes, and plant parts can be screened to maximize the antioxidant properties of *A. chinensis*.

### Anti-Inflammatory Activity

Anti-inflammatory activity of *A. chinensis* has been proved *in vivo* and *in vitro* models. On high-fat diet-induced obese C57BL/6 mice models, consecutive consumption the seeds oil of *A. chinensis* with 1.0 and 3.0 ml/kg·bw ameliorated obesity-induced inflammation by down-regulating the mRNA expression of related to inflammation adipokines, such as TNF-α, IL-6, IL-1β, COX-2, and iNOS (Qu et al., 2019). The aqueous and ethyl acetate extracts demonstrated anti-inflammatory activity in inflammatory bowel disease models of the *IL-10* gene-deficient mice (Edmunds et al., 2012). In patients with type-2 diabetes mellitus, the fruit juice of *A. chinensis* showed preventative activity on inflammation by activating Keap1 and Nrf2 *via* upregulating miR-424 (Sun et al., 2017). On the cellular level, polyphenols mainly composed of protocatechuic acid, *p*-hydroxybenzoic acid, *p*-coumaric acid, caffeic acid, and ferulic acid from seeds of *A. chinensis* at concentration of 40 and 60 μg/ml for 12 h decreased the secretion of pro-inflammatory cytokines IL-1β and TNF-α in LPS-induced RAW 264.7 cells (Deng et al., 2016). Therefore, the anti-inflammatory potential *A. chinensis* seeds mainly depend on the synergetic effect of these polyphenols, and it may be used to prevent a variety of inflammation related diseases.

### Antibacterial Activity

All the extracts including skin, pulp, seeds, and stems showed bactericidal against Staphylococcus aureus, Streptococcus pyogenes, S. faecalis, Salmonella typhi, Proteus mirabilis, Pseudomonas aeruginosa, Escherichia coli, and Klebsiella pneumonia. The skin and pulp extracts showed inhibition activity against S. aureus and S. pyogenes with MIC values of 8 and 4 μg/ml, but they showed moderate inhibition activity against S. faecalis, S. typhi, P. mirabilis, P. aeruginosa, E. coli, and K. pneumonia with MIC values ranging from 16 to 128 μg/ml. The leaves and stems extract just inhibited S. pyogenes and P. aeruginosa with MIC values of both 64 and 32 μg/ml. The seeds extracts showed an exclusively bacteriostatic activity against these selected strains of bacteria with MIC values of between 1 and 8 μg/ml (Basile et al., 1997). Polyphenol from seeds of A. chinensis showed significant bactericidal against Bacillus cereus, B. subtilis, Shigella flexneri, and Salmonella Typhi, and bacteriostatic against B. thuringiensis. We can find that the antimicrobial activity of the polyphenol extract on gram-positive bacteria is higher than that of gram-negative bacteria (Deng et al., 2013). Therefore, kiwifruit seeds are potential food processing material for their antimicrobial activity.

### Immunoregulatory Activity

Consumption of the aqueous extracts of whole fresh fruit of Hort16Aat 375 mg/kg for 12 days enhanced both innate and acquired immunity in cholera vaccine and tetanus/diphtheria vaccine models in Balb/c mice, showing a beneficial effect on healthy (Shu et al., 2008). The homopolysaccharide derivatived by O-sulfation from the roots of *A. chinensis* at concentration of 10 and 50 μg/ml activated phagocytic activity and increased NO production of RAW 264.7 macrophages, and the activity of sulfated polysaccharides is strongly related to the degree of the sulfation (Niu et al., 2016), and treatment with 50-300 μg/ml water-soluble polysaccharides dose-dependently stimulated NO production and phagocytic activity of RAW 264.7macrophages (Zhang et al., 2015). It remains to clarify the detailed mechanism of immunoregulatory activity and the responsible compositions for this valid action.

### Hypolipemic and Antidiabetic Activities

Administered the seed oil of *A. chinensis* rich in fatty acids at 1.0 and 3.0 ml/kg·bw daily over 12 consecutive weeks significantly lowered bodyweight gain, inguinal fat tissue weight, and the accumulation of TC, TG, HDL-C, and LDL-C in liver of the high-fat diet-induced obese C57BL/6 mice. Meanwhile, long-term consumption of the seed oil of *A. chinensis* up-regulated the expression of thermogenesis-related genes like *PPAR*-γ, *UCP1*, *PGC1*-α, and *PRDM16*, down-regulated FAS expression, and altered the gut microbiota by decreasing the *Firmicutes*-to-*Bacteroidetes* ratio (Qu et al., 2019). In addition, the seed oil from *A. chinensis* supplementation improved insulin resistance and alleviated hyperglycemia by reducing HOMA-IR index and blood glucose in high fat diet-induced obese mice (Qu et al., 2019). Thus, the lipid lowering potential of *A. chinensis* seed provide a basis theory for food industries.

### Cardiovascular Protective Effects

In H9c2 rat cardiac myocytes cells induced by hypoxia in cardiomyocytes treated with angiotensin II, treatment with 1.25 and 2.5 mg/ml polysaccharide of *A. chinensis* alleviated cardiac hypertrophy, decreased mitochondrial dysfunction and reduced cardiomyocytes apoptosis by decreasing the apoptosis-associated genes expression like *mitochondria associated-1* and *caspases3/8/9*, and cleaving caspases-3/8/9. Additionally, the protective effects of polysaccharide against hypoxia-induced apoptosis may be attributable to inactivate the ERK1/2 and PI3K/AKT signaling pathways (Wang et al., 2018). The polysaccharide of *A. chinensis* can be potentially used in the treatment of heart disease. However, it is noteworthy that polysaccharide at high dose (10 mg/ml) suppressed the cardiomyocytes viability.

### Hypnotic Effects

Oral administration of ethanol extracts from *A. chinensis* peel at dose of 250, 500, and 1,000 mg/kg dose-dependently decreased sleep latency and increased sleep duration in pentobarbital-treated mice. Especially, the sequentially partitioned with ethyl acetate fraction rich in flavonoids (1.63 mg QE/g) at 250 mg/kg exert significantly hypnotic effects and this sedative-hypnotic activity could be inhibited by GABA_A_-BZD receptor antagonist flumazenil. The flavonoids may be attributable to hypnotic activity *via* allosteric GABA_A_-BZD receptor modulation, but the precise mechanisms and the existing individual flavonoids are needed to be evaluated in the future (Yang et al., 2013).

### Ace Inhibitory Activity

The 70% aqueous acetone extracts partitioning with hexane rich in flavonoid from Hort 16A dose-dependently inhibited ACE activity with IC_50_ of 12.81 mg/ml using a fluorescence-based biochemical assay. LC-MS/MS showed that the higher total phenolic and total flavonoid contents are identified in this extract. UPLC-MS/MS showed that polyphenols (231.32 µg/g DW) in the extract are mainly flavonols, flavanols, and phenolic acids. Specifically, quercetin-3-O-galactoside (205.19 µg/g DW), quercetin-3-O-glucoside (0.45 µg/g DW), quercetin-3-O-rhamnoside (0.61 µg/g DW), quercetin-3-O-rutinoside (0.29 µg/g DW), epicatechin (5.15 µg/g DW), catechin (0.75 µg/g DW), epigallocatechin (0.61 µg/g DW), phloridzin (2.03 µg/g DW), and isoferulic acid (15.12 µg/g DW) are major compounds in the extract (Hettihewa et al., 2018). These compounds could be responsible for the observed *in vitro* ACE inhibitory activity of Hort 16A fruit, though the active compounds identifying and *in vivo* animal studies remain to be investigated and conducted.

### Dermatological Activity

The raw polysaccharides with >90% carbohydrate and 5.2% residual protein from the fresh fruit of *A. chinensis* at 10 μg/ml showed a significantly proliferation-promoting on cell proliferation rates of HaCaT cell line and primary keratinocytes (NHK), and it also significantly promoted proliferation of human dermal fibroblasts at 132 and 198 μg/ml. Meanwhile, treatment of the polysaccharides at 200 μg/ml significantly stimulated ATP-synthesis, promoted mitochondrial activity and energy metabolism of HaCaT keratinocytes, and significantly increased collagen synthesis in dermal skin equivalents (Deters et al., 2005). Kiwifruit pericarp proanthocyanidins mainly contained B-type propelargonidins, procyanidins, procyanidins gallate, and prodelphinidins showed strongly inhibition activity on tyrosinase, indicating that it can be used as whitening agents (Chai et al., 2014).

### Cytochrome P450 Enzyme Inhibitory Activities

Cytochrome P450 system in liver plays an important role in drug metabolism. It transforms drug from hydrophobic to hydrophilic, which is easier to excrete. The 90% EtOH extract of *A. chinensis* root at 50 μg/ml exhibited inhibition activities on CYP2C9, CYP2D6, and CYP3A4 in human liver tissue with the 69.0, 76.3, and 53.3% of remaining activity, respectively. The inhibitory effect of the crude extract could be largely attributed to the presence of triterpenoids (Xu et al., 2016). It is worth noting that the combination of crude extracts or these triterpenoids with other medical herbs or drugs may lead to drug interaction with cytochrome CYPs at pharmacokinetic and pharmacodynamic levels, which indicates that people should cautiously consume *A. chinensis* fruit when taken medicine.

### Processing and Utilization

Chinese kiwifruit is a very high nutritional value of nourishing and consumers’ favorite fruit, which has shown application potential in food, medicine, and health products industry. China is the largest kiwifruit producer in the world. In 2016, kiwifruit production in China reached 2.41 million tons per year, accounting for 56.0% of the world’s total kiwifruit production (United Nations Food and Agriculture Organization, 2016). To date, a series of commercially available products has been processed due to abundant nutrient substance and claimed health benefits. These Chinese kiwifruit related products include sliced fruit, juice, preserved fruit, yogurt, wine, canned fruit, dried kiwi slices, fruit vegetable juice drinks, biscuits, milk beverage, whipped cream, baked goods, vinegar, and oil capsule. Furthermore, various different parts of *A. chinensis* showed different uses. Briefly, the leaves contain protein, starch, and polyphenols, which may be developed as an excellent source of natural products. The beautiful and fragrant of Chinese kiwifruit flowers rich in honey juice and volatiles can be used as high-quality honey source. Kiwifruit peel residue as sources of high-quality pectin can be used as functional ingredient for food products. Chinese kiwifruit seeds rich in essential fatty acids, protein, and dietary fiber can be used in food and health products industry (Xie, 1975; Garcia et al., 2012). The roots and barks contain ursolic acid, oleanolic acid, and quercetin, which have antitumor effect against liver cancer, lung cancer, gastric cancer, esophageal cancer, colorectal cancer, and cervical cancer (Chang and Case, 2005; Xu et al., 2010; Wei et al., 2018). The different parts of *A. chinensis* are widely used as pharmaceutical raw materials in medicine for prevention and treatment of tumors. In addition, the various claimed nutritional and pharmacological properties including strong antitumor, antioxidation, and anti-inflammatory potential of various extracts or active compounds of *A. chinensis* indicated that they could be further developed for functional food with added-commercial value or effective and safe drug formulations.

### Storage Methods

Chinese kiwifruit has a short postharvest life because of fast softening and serious decay. Preservation of Chinese kiwifruit for prolonged periods is particularly important. Freezing and frozen storage is currently the most common method, which can effectively inhibit the softening of kiwifruit and prolong its postharvest life. However, kiwifruit is cold-sensitive and very susceptible to chilling injury when storage at the temperature between −2°C and 2.5°C for a long time (Gerasopoulos et al., 2006; Ma et al., 2014). Interestingly, dipped by water for 10 min at 45°C to low temperature storage can prevent chilling injury development to kiwifruit. Meanwhile, the kiwifruit pretreated at 45°C and then stored at 0°C for 90 days showed higher firmness and soluble solids content, and MDA content and lipoxygenase activity in kiwifruit are reduced. However, pretreated at 20 and 55°C were ineffective at alleviating chilling tolerance (Ma et al., 2014). Various other treatments including preharvest calcium chloride sprays (Gerasopoulos and Drogoudi, 2005), putrescine (Yang et al., 2016), preharvest chilling (Sfakiotakis et al., 2005), and gradual cooling (Yang et al., 2013) have also been used to alleviate chilling injury in kiwifruit.

After harvest, kiwifruit is highly perishable, and its nutritional ingredients and quality decline rapidly due to the influence of internal biochemical reactions and external environment. The modified atmosphere packaging, chitosan, 1-methylcyclopropene, ClO_2_, ozone, tea polyphenols, protein, lipid composite film, oxalate, salicylic acid, and citric acid have been used individually or combined to alleviate physicochemical quality changes for postharvest of kiwifruit (Huang et al., 2017). The ozone treatment induced the ripening process, delayed the microbial growth, and influenced the content of vitamin C, polyphenols, flavonoids, and carotenoids (Goffi et al., 2019). The chitosan combined with salicylic acid treatment during storage at room temperature for 14 days provides a significantly effective preservative effect by delayed vitamin C and soluble solids decomposition, inhibiting moisture loss and acidity change, and maintaining texture and surface color of Chinese kiwifruit in 14 days of storage at room temperature (Huang et al., 2017).

## Conclusions

Chinese kiwifruit and related products are increasingly popular throughout the world due to the remarkably economic, nutritional, and health benefits values. It is a good source of phenolic compounds, vitamin C, carbohydrates, sugars, amino acids, and minerals. Of particular note in kiwifruit is vitamin C and minerals K. The phenolic compounds present in Chinese kiwifruit are organic acids and flavonoids, and fruit peel and flesh, leaf, vine, and roots also contain a variety of these phenolic components. The major components of the roots are triterpenoids characterized by 12-en-28-oic acids of oleanane and ursane type. Terpenes, straight chain alkenes, alcohols, and esters were dominant volatile components in flowers and roots of *A. chinensis*. These chemical compounds render the *A. chinensis* with a range of sensory quality, nutritional, and pharmacological properties as proved by *in vitro* and *in vivo* studies. The claimed biological activity of isolated compounds, fractions, or crude extracts include antitumor, antioxidant, anti-inflammatory, antibacterial, immunoregulatory, hypolipemic, antidiabetic, and cardiovascular protective effects. Of particular note is that these claimed biological activities such as antitumor, antioxidant, and immunoregulatory may be greatly attributed to the existence of triterpenoids, polyphenols, flavonoid, polysaccharide, unsaturated fatty acid, and vitamin C. These findings suggest that Chinese kiwifruit can be useful in the prevention and treatment of pathologies associated to cancer, oxidative stress, and aging.

There are also research opportunities to better development, utilization, and protection kiwifruit for human consumption. Cytochrome P450 inhibitory activities, toxicity analysis, qualitative and quantitative metabolite research, effective and standardized quality standard building, and clinical studies should be encouraged to conducted for safe daily consumption. Meanwhile, the synergism and attenuation effects, metabolic behavior of various ingredients, as well as the *in vivo* and molecular mechanisms studies responsible for the observed biological properties should be conducted. It is also found that some of the *A. chinensis* cultivars were only supported by a few studies, and confirmative studies should be conducted to verify their health effects. Apart from the fruit, other plant parts of kiwifruit including leaves and roots should also be explored for effective utilization. The effective method and technology for the storage and preservation of kiwifruit during preharvest and postharvest remain to be explored to avoid the frequent chilling damage, soft rot, and mildew, and also decrease and improve the change of the chemical profile and bioactivity properties during storage.

## Author Contributions

XC and YM obtained the literatures. JF, ZZ, and XH wrote the manuscript. XH, LH, and YL gave ideas and edited the manuscript. All authors approved the paper for publication.

## Conflict of Interest

The authors declare that the research was conducted in the absence of any commercial or financial relationships that could be construed as a potential conflict of interest.

## Abbreviations

A549, Human alveolar basal epithelial cells; ABTS, 2′-azino-bis (3-ethylbenzthiazoline-6-sulfonic acid; ACE, Angiotensin converting enzyme; ALT, Alanine aminotransferase; AST, Aspartate aminotransferase; CE, Catechin equivalents; COX-2, Cyclooxygenase-2; CYP2C9, Cytochrome P450 2C9; CYP2D6, Cytochrome P450 2D6; CYP3A4, Cytochrome P450 3A4; CYPs, Cytochrome P450; DLX2, Distal-less-like homeobox protein; DPPH, 2,2-diphenyl-1-picrylhydrazyl; DW, Dry weight; EP3, Prostaglandin E2 receptor subtype 3; ERK1/2, Extracellular regulated protein kinases; FAS, Fatty acid synthase; FDW, Freeze-dried weight; FRAP, Ferric ion reducing antioxidant power; FW, flesh weight; GABA-BZD, γ-aminobutyric acid-benzodiazepine; GAE, Gallic acid equivalents; GC-MS, Gas chromatography-mass spectrometer; GSH, Glutathione; HaCaT, Human immortalized keratinocytes; HCC, Hepatocellular carcinoma; HDL-C, High density lipoprotein cholesterol; HEK 293, Human embryonic kidney 293 *cells*; HepG2, Liver hepatocellular cells; HO·, Hydroxyl radical; HOMA-IR, Homeostasis model assessment for insulin resistance; HPLC, High-performance liquid chromatography; IC_50_, Half maximal inhibitory concentration; IL-1β, Interleukin-1β; IL-6, Interleukin-6; iNOS, Inducible nitric oxide synthase; Keap1, Kelch-like ECH-associated protein 1; LC-MS/MS, Liquid chromatography-*tandem* mass spectrometry; LDL-C, *Low density lipoprotein cholesterol*; LoVo, Colorectal cancer cell line; MCF-7, Human breast adenocarcinoma cell line; MDA, Malondialdehyde; MIC, Minimum inhibitory concentration; NCI-H460, Large cell lung cancer cells; NF-κB, Nuclear factor-kappa B; NO, Nitric oxide; Nrf2, Nuclear factor (erythroid-derived 2)-like 2; ORAC, Oxygen radical absorbance capacity; P-388, Mouse leukemia cells; PGC1-α, Peroxisome proliferator-activated receptor gamma coactivator 1-alpha; PI3K/AKT, Phosphatidylinositol 3-kinase/protein kinase B; PPAR-γ, Peroxisome proliferators-activated receptors; PRDM16, PR domain containing 16; QE, Quercetin equivalents; RAW 264.7, Mouse leukaemic monocyte macrophage cell line; SK-OV-3, Human ovarian epithelial cancer cells; SOD, Superoxide dismutase; TC, Total cholesterol; TG, Triglyceride; TNF-α, Tumor necrosis factor alpha; UCP1, Uncoupling protein 1; UPLC-MS/MS, Ultra-high-performance liquid chromatography-tandem mass spectrometry; UPLC-QqQ-MS/MS, Ultra-high-performance hydrophilic interaction liquid chromatography-triple quadrupole tandem mass; UPLC-TOF/MS, Ultra-performance liquid chromatography quadrupole-time of flight mass spectrometry; USDA, United States Department of Agriculture; UV/Vis, Ultraviolet-visible spectroscopy; VCE, Vitamin C equivalents; VEGFR2/Src/FAK, Vascular endothelial growth factor receptor 2/Src/focal adhesion kinase.
